# Investigation of the Melt-Rotation Effects on Fiber Orientation Variation and Geometrical Shrinkage in FRP Injection-Molded Parts

**DOI:** 10.3390/polym17172360

**Published:** 2025-08-30

**Authors:** Jing-Kai Gao, Fang-Lin Hsieh, Min-Yuan Chien, Chao-Tsai Huang

**Affiliations:** 1 Department of Chemical and Materials Engineering, Tamkang University, New Taipei City 251301, Taiwan; kyle900425@gmail.com (J.-K.G.); dick64049@gmail.com (F.-L.H.); 2 Department of Vehicle Engineering, Chien Hsin University of Science and Technology, Taoyuan City 320678, Taiwan; jackyaren@uch.edu.tw

**Keywords:** injection molding, fiber reinforced thermoplastics (FRPs), fiber orientation, melt rotation

## Abstract

The study focuses on the asymmetric shrinkage typically occurring between the upstream and downstream regions of FRP injection-molded products, a challenge that is particularly difficult to manage and improve. Specifically, two sets of four-cavity systems in one mold were utilized as the experimental platform. One set used a balanced runner (BR) system, and the other used a non-balanced runner (NBR) system. Each cavity in the four-cavity systems contained an ASTM D638 standard specimen with dimensions of 63.5 mm × 9.53 mm × 3.5 mm. Both CAE simulation and experimental methods were applied. The results show that the filling patterns from the simulation analysis closely matched those from the experimental study for both BR and NBR systems. Furthermore, by comparing the geometric shrinkage of the injected parts, significant differences were observed in the dimensional deformation in three directions (*x*, *y*, and *z*) between the NBR and BR systems. Specifically, at the end of the filling region (EFR), there was no noticeable difference in shrinkage along the flow direction, but the shrinkage in the cross-flow and thickness directions was reduced in the NBR system. Additionally, for the same cavity (1C) in both BR and NBR systems, the melt-rotation effect significantly reduced shrinkage in both the cross-flow and thickness directions. These findings strongly suggest that melt rotation can effectively modify the dimensional shrinkage of injection-molded parts. Moreover, fiber orientation analyses of the 1C cavity were also performed using CAE simulation for both BR and NBR systems. The results show that in the NBR system, the melt-rotation effect substantially alters the fiber orientation. Specifically, the fiber orientation tensors in the cross-flow (A_22_) direction exhibit a decreasing trend. It can be speculated that the melt rotation alters the flow field, which subsequently changes the fiber orientation by reducing the flow-fiber coupling effect, thereby reducing the upstream-to-downstream asymmetry in the cross-flow direction. Through in-depth analysis, it is demonstrated that the correlation between the macroscopic geometric shrinkage and the microscopic fiber orientation changes is highly consistent. Specifically, in the EFR, ΔA_22_ decreased by 0.0376, improving upstream/downstream shrinkage asymmetry in the cross-flow direction (Ly). Future work will investigate alternative melt-rotation designs and the optimization of model-internal parameters in FOD prediction.

## 1. Introduction

FRPs have gained significant attention in the production of lightweight vehicle body components [1,2], renewable energy equipment [3,4], and even in new space components [5] owing to their exceptional mechanical properties. Numerous studies have been conducted to explore how fiber reinforcement enhances the properties of plastics. Thomason [6,7] provided empirical guidelines to assist the industry in determining the minimum fiber length required for auto parts to achieve adequate rigidity and impact strength. Other researchers [8,9] have investigated methods for further improving mechanical properties by combining various materials and formulations. Additionally, a considerable body of research [10,11,12,13,14,15,16] has examined the effects of process-induced changes in fiber microstructure. For instance, Rohde et al. [10] employed thermal degradation techniques to assess the influence of processing parameters on fiber length variation, while Goris et al. [11] introduced the PEC technique to evaluate post-injection-molding fiber length, identifying screw speed and back pressure as critical factors influencing fiber breakage. However, studies [11,12] have highlighted that measuring small fiber quantities in each sample can lead to significant inaccuracies, emphasizing the need for larger experimental datasets to obtain reliable results in fiber breakage investigations. This requirement presents a considerable challenge in the field. Several researchers [13,14,15] have used optical and tomographic techniques to examine fiber microstructure in injected FRP components. Bernasconi et al. [13] found that the classical optical sectioning method may not always provide accurate fiber orientation measurements and emphasized the need to select the correct section plane. When properly chosen, this method, though destructive, can yield results comparable to the nondestructive micro-CT method. Gandhi et al. [14] used micro-CT to capture 3D images of fibers and analyze their orientation, noting the challenge of handling large image data. By applying image technologies to reduce data size, they found that micro-CT results were similar to the conventional ellipse method. Teuwsen et al. [15] employed micro-CT and VG Studio analysis to investigate fiber orientation and concentration, revealing nonuniform patterns along the part thickness and flow path, with a distinct seven-layered fiber orientation pattern. Huang et al. [16] used micro-CT and Avizo analysis to explore the relationship between fiber orientation distribution (FOD) and tensile stress, showing that side gate design improves fiber orientation distribution and mechanical strength. The literature suggests that the superior performance of FRPs over general plastics is primarily due to their fiber microstructural properties, such as orientation, length, and concentration, which strongly influence mechanical properties. Furthermore, Quintana et al. [17] demonstrated that anisotropic CAE models can effectively capture the overall trends of fiber alignment, although their accuracy remains limited at the micro-scale. They further emphasized that integrating micro-CT with CAE significantly improves the reliability of FOD prediction tools in injection molding. Despite various nondestructive and destructive techniques, understanding fiber reinforcement mechanisms through experimental methods remains challenging.

To further understand the changes in fiber microstructure during the injection-molding process, Folgar and Tucker [18] and Advani and Tucker [19] developed theoretical models to predict the fiber orientation of short fibers in FRP injection molding. These models have become valuable tools for guiding the design and advancement of FRP injection products in both industry and academia [20,21]. However, Thomason [6,7] stressed the critical importance of maintaining an adequate fiber length in the final product to meet impact strength requirements for practical applications. A significant challenge lies in effectively integrating long fibers into the polymer matrix and preserving their length after injection, whether approached through theoretical predictions or experimental observations. To address this, scholars have introduced the Anisotropic Rotary Diffusion (ARD) model [22] and the Reduced Strain Closure (RSC) model [23,24], which provide deeper insights into the microstructural properties of long fiber-reinforced plastics. While these numerical models are valuable, they require numerous additional parameters and are complex to implement. In response, Tseng et al. [25] simplified the model parameters through the iARD-RPR model, which was subsequently integrated into the commercial software Moldex3D. Moreover, although CAE simulations facilitate FOD prediction, the large number of parameters embedded in most models poses significant challenges for their accurate determination, presenting ongoing difficulties for both industrial practice and academic research. Żurawik [26] evaluated the Moldflow Rotational Diffusion (MRD) model for FOD prediction and, using micro-CT validation, showed that it provides reasonable accuracy but still deviates in high-shear and EFR. The study highlighted µCT as a reliable benchmark for validating FO models. Rienesl [27] used particle swarm optimization (PSO) to calibrate model parameters with micro-CT data, demonstrating improved FOD prediction accuracy and underscoring the importance of micro-CT for parameter tuning. According to the above literature, obtaining and optimizing numerous internal model parameters is crucial for accurate simulation analysis of FRP injection molding.

During the injection-molding process, molten polymer flow significantly influences fiber orientation, while fibers also affect the flow of the polymer melt. This interaction, known as the flow–fiber coupling effect, has been widely studied [28,29,30,31,32]. Lipscomb et al. [28] introduced a constitutive equation to analyze the flow–fiber coupling effect in an axisymmetric contraction flow system, finding that stronger coupling leads to a larger corner vortex. VerWeyst and Tucker [29] expanded this research by introducing the concept of Np (particle number) to quantify the coupling effect. Their 2.5D simulation showed that higher Np had minimal effect on streamlines and velocity profiles, with little impact on fiber orientation. Tseng and Su [30] extended this work into 3D simulations, showing that while increasing Np had little impact on velocity and fiber orientation, high Np values (20) caused computation divergence. To address the limitations of the Lipscomb equation, Favaloro et al. [31] proposed the IISO viscosity model to account for stress tensor variations based on fiber orientation. Their research showed plug flow behavior and provided insights into fiber orientation in the flow direction (A_11_). Tseng and Favaloro [32] refined the IISO model, observing a transition in flow front behavior from convex–flat–flat to convex–flat–concave, influenced by coupled flow and fiber interactions. Despite these advancements, practical verification remains limited. Building on their work, Huang and Lai [33] confirmed the convex–flat–concave flow front behavior in a more complex center-gated plate, showing that the flow–fiber coupling effect was more prominent at EFR and reduced in the near-gate region (NGR) due to strong shear forces, as verified by simulations and experiments.

Traditionally, polymer melt is conveyed through conventional runner systems, which lack effective adjustability within the mold cavity. However, existing literature indicates that modifying the flow path can potentially reverse the polymer melt’s direction, altering the melt-flow characteristics and ultimately affecting product quality. Notably, Beaumont and colleagues [34,35,36] extensively investigated this phenomenon and developed the commercial product MeltFlipper^®^.(supplied by Beaumont Technologies, Inc., Erie, PA, USA). Initially, when the polymer melt flowed through geometrically balanced runner systems, the resulting product deviated from the expected outcome. The researchers attributed this discrepancy to the shear heat generated during melt flow, prompting them to introduce a melt flip design element in the flow channel to counteract the unbalanced polymer melt flow. Later, Chien et al. [37,38] employed the finite volume method to simulate polymer melt behavior in cold runner systems, revealing that flow imbalances arose from shear-induced heat generation, which caused temperature gradients and viscosity changes in the material, ultimately impacting product quality. They also demonstrated that melt flipping rearranges the flow field, further influencing melt flow. Moreover, Petzold et al. [39] proposed methods to optimize the temperature field in hot runner systems, while Wilczyński et al. [40] introduced strategies for addressing filling imbalances in geometrically balanced mold systems, highlighting the effectiveness of the artificial neural network (ANN) method as an optimization tool. Recently, Shotwell et al. [41] showed that helical static mixers in the runner can temporarily randomize fiber orientation near the gate, with fibers re-aligning downstream, suggesting their potential for controllable anisotropy management. Land et al. [42] demonstrated that using a rotating mold core to impose melt rotation can effectively control fiber orientation, with gate design and wall thickness influencing its benefits. In addition, Hsieh et al. [43] showed that runner symmetry and overflow features affect flow–fiber coupling and shrinkage asymmetry, offering design strategies to control FOD. The reviewed studies indicate that FOD can be manipulated by adjusting the melt flow via screw and runner design. Nevertheless, shear-induced variations result in distinct upstream and downstream orientation patterns, and robust approaches for qualitative or quantitative control have yet to be established.

Based on the literature reviewed above, FRP injection molding is widely applied across industries, offering substantial benefits in enhancing mechanical properties and enabling lightweight design. However, the highly anisotropic FOD induced during processing often results in asymmetric shrinkage between upstream and downstream regions of the molded product, posing significant challenges for industrial applications, particularly in products requiring stringent dimensional precision. Although this asymmetric shrinkage is generally attributed to fiber orientation, the exact nature of their relationship remains insufficiently understood. Moreover, no definitive method has yet been established to effectively manipulate fiber orientation within the melt to mitigate asymmetric shrinkage in these regions. Hence, this study introduces a melt-rotation structure design to investigate, through simulation and experimental validation, how melt rotation influences injection molding. The objectives are as follows: (1) examine its effect on product geometry, (2) assess its impact on internal fiber orientation, and (3) clarify the correlation between geometric dimensions and fiber orientation.

**Figure 1:**
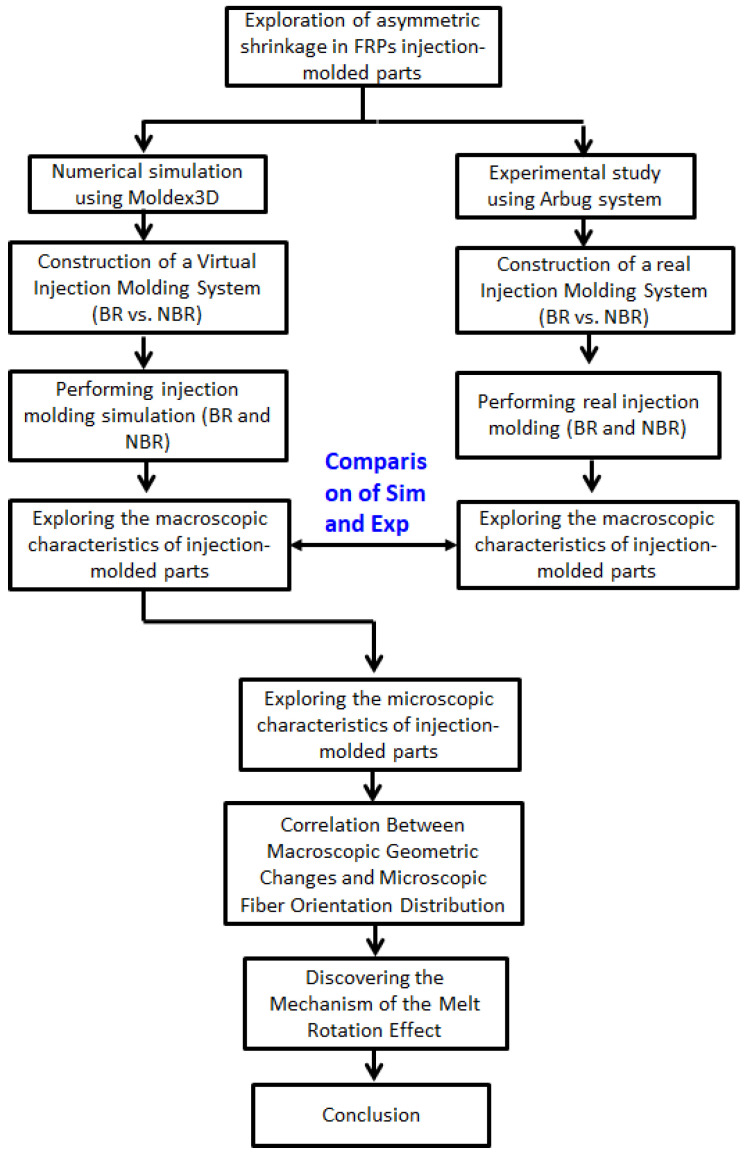
Flow chart of this study.

To achieve the objectives, and based on the author’s experience, when product design and mold design remain unchanged, merely adjusting the operating conditions of conventional injection molding cannot effectively address the asymmetric shrinkage problem of FRP injection products. Therefore, this study builds on the concepts presented in references [34,35,36,37,38,40,42,43] and introduces a melt-rotation structure designed to alter the melt behavior within the flow channel. This approach aims to modify both the internal fiber orientation distribution (microscopic properties) and the external geometric shrinkage characteristics (macroscopic properties) of the final product. The investigation of these effects is integrated with CAE simulation and experimental validation to establish a digital twin system. The relevant technologies and processes are primarily based on reference [43]. Specifically, the study applies polypropylene (PP) and PP containing 30 wt% short glass fiber (30SFPP) materials. Two sets of 1 × 4 multi-cavity mold systems are used to prepare injection-molded products for research. One set utilizes a balanced runner (BR) system, while the other features a non-balanced runner (NBR) system with a special melt-rotation design. To gain a deeper understanding of the melt rotating effect and its impact, this study employs both theoretical simulation analysis and experimental research. Specifically, it explores changes in melt flow within the mold cavity, thoroughly analyzing how the melt-rotation effect influences macroscopic geometric edge length shrinkage in injection-molded products. Additionally, the study also investigates how the melt rotating effect affects microscopic fiber orientation changes in the product. Finally, it examines the correlation between these microscopic fiber orientation changes and the macroscopic geometric edge length shrinkage. The details are illustrated in the flow chart shown in Figure 1 and are further described in the following sections.

## 2. Theory and Mathematical Models

### 2.1. Injection-Molding Polymer Processing

During injection molding, polymer melts, regardless of the presence of reinforcing fibers, behave as compressible, non-Newtonian fluids. Consequently, their flow must be described by the three-dimensional, transient, and non-isothermal governing equations of fluid mechanics [14,24]:

(1) $$\frac{\partial \rho }{\partial t}+\nabla \cdot \rho u=0,$$

(2) $$\frac{\partial }{\partial t}\left(\rho u\right)+\nabla \cdot \left(\rho uu\right)=\nabla \cdot \sigma +\rho g,$$

(3) σ=−PI+τ,

(4) $$\rho C_{P}\left(\frac{\partial T}{\partial t}+u\cdot \nabla T\right)=\nabla \cdot \left(k\nabla T\right)+\tau :D,$$

where ρ is density; t is time; **u** is the velocity vector; **σ** is the total stress tensor; **g** is the acceleration vector of gravity; **τ** is the extra stress tensor; *P* is pressure; C_P_ is specific heat; T is temperature; k is thermal conductivity; and **D** is the rate-of-deformation tensor.

Moreover, for polymer melts, the extra (deviatoric) stress tensor, **τ**, is typically expressed as

(5) τ=2ηD,

where η is the shear viscosity of a polymer melt.

To quantify the viscosity of the polymeric melt as a function of temperature, we apply the modified Cross model, formulated as the following equation. In this expression, **η_0_** is the zero-shear (Newtonian-plateau) viscosity, **n** is the power-law index, and τ* is the characteristic shear stress that marks the transition from the zero-shear (viscosity-plateau) region to the shear-thinning power-law region of the viscosity curve. In addition, *B* is a pre-exponential factor. It is typically obtained by fitting experimental rheological data to the modified Cross model.

(6) $$\eta \left(T,\dot{\gamma }\right)=\frac{\eta_{o}\left(T\right)}{1+{\left(\eta_{o}\dot{\gamma }/\tau^{*}\right)}^{1-n}},$$

(7) $$\eta_{o}\left(T\right)=\mathrm{BExp}(\frac{T_{b}}{T})$$

### 2.2. Fiber Orientations Inside the Polymeric Matrix

During the injection molding of FRPs, every embedded fiber is idealized as an axisymmetric rigid rod. The orientation of an individual rod is specified by the unit vector **p** directed along its axis. The ensemble orientation of many fibers is succinctly captured by the second-order orientation tensor **A**:

(8) $$A=\oint \psi \left(p\right)pp\;dp,$$

where ψ(p) is the probability density distribution function over orientation space. Tensor **A**_4_ is a fourth-order orientation tensor, defined as follows:

(9) $$A_{4}=\oint \psi \left(p\right)pppp\;dp.$$

To address the complexity of the orientation tensor evolution, Tseng et al. [24] enhanced the anisotropic rotary diffusion–reduced strain closure (ARD–RSC) framework [21,22,23] and coupled it with Jeffery’s hydrodynamic theory, producing the inhomogeneous iARD–RPR model, described in Equation (10). In this formulation, $\dot{A}$ is the material (substantial) derivative of the orientation tensor A. The coefficients C_I_ and C_M_ account for fiber–fiber and fiber–matrix interactions, respectively, while the damping parameter α retards the rate at which the fiber orientation adapts to the local flow.

(10) $$\dot{A}={\dot{A}}_{HD}+{\dot{A}}_{iARD}\left(C_{I},\;C_{M}\right)+{\dot{A}}_{RPR}(\alpha ),$$

Moreover, Jeffery’s hydrodynamic (HD) term can be expressed as follows:

(11) $${\dot{A}}_{HD}=\left(W\cdot A-A\cdot W\right)+\xi (D\cdot A+A\cdot D-2A_{4}:D),$$

(12) $$D=\frac{1}{2}\left(\nabla u+\nabla u^{T}\right),$$

(13) $$W=\frac{1}{2}\left(\nabla u-\nabla u^{T}\right),$$

where **W** and **D** are the vorticity tensor and the rate-of-deformation tensor, respectively. ξ is a shape factor of a particle. The rest of the details for the RPR model and the iARD model are available elsewhere [24].

### 2.3. Revised Viscosity by Flow–Fiber Coupling

To capture the complex flow–fiber interaction, we use the updated IISO constitutive framework with an iterative solution strategy. First, the 3D flow and fiber orientation fields are solved using a finite volume scheme. The resulting velocity and orientation tensors are then used to compute an updated IISO viscosity. This process is repeated until the velocity, pressure, and viscosity fields converge. A detailed algorithm description is available in [29]. The modified IISO viscosity, incorporating the current fiber orientation, is given by:

(14) $$\eta^{IIOS}=(1+R_{T}K_{s\;})\eta_{S},$$

(15) $$R_{T}\left(\dot{\gamma }\right)=\frac{R_{T}^{0}}{1+{\left(\dot{\frac{\dot{\gamma }}{{\dot{\gamma }}_{C}}}\right)}^{2}},$$

(16) $$K_{s}=\frac{D:A_{4}:D}{2D:D},$$

where $\eta_{S}$ is the nonlinear Newtonian viscosity for the fiber-filled polymer fluids and is described by the modified Cross model; *R_T_* is the dimensionless Trouton ratio parameter as a function of the strain rate; $R_{T}^{0}$ is the initial value of *R_T_*; *K_S_* is a stretching kernel that is related to the flow fields and the fiber orientation state; and ${\dot{\gamma }}_{C}\;$ is the critical strain rate (1/s).

## 3. Systems and Information

### 3.1. Simulation System and Related Information

**Figure 2:**
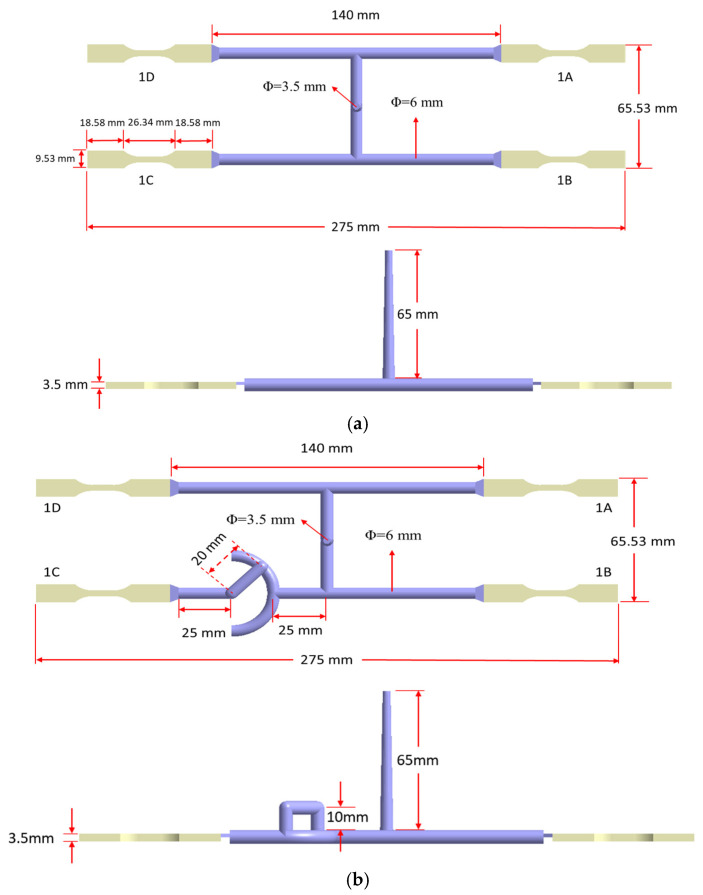

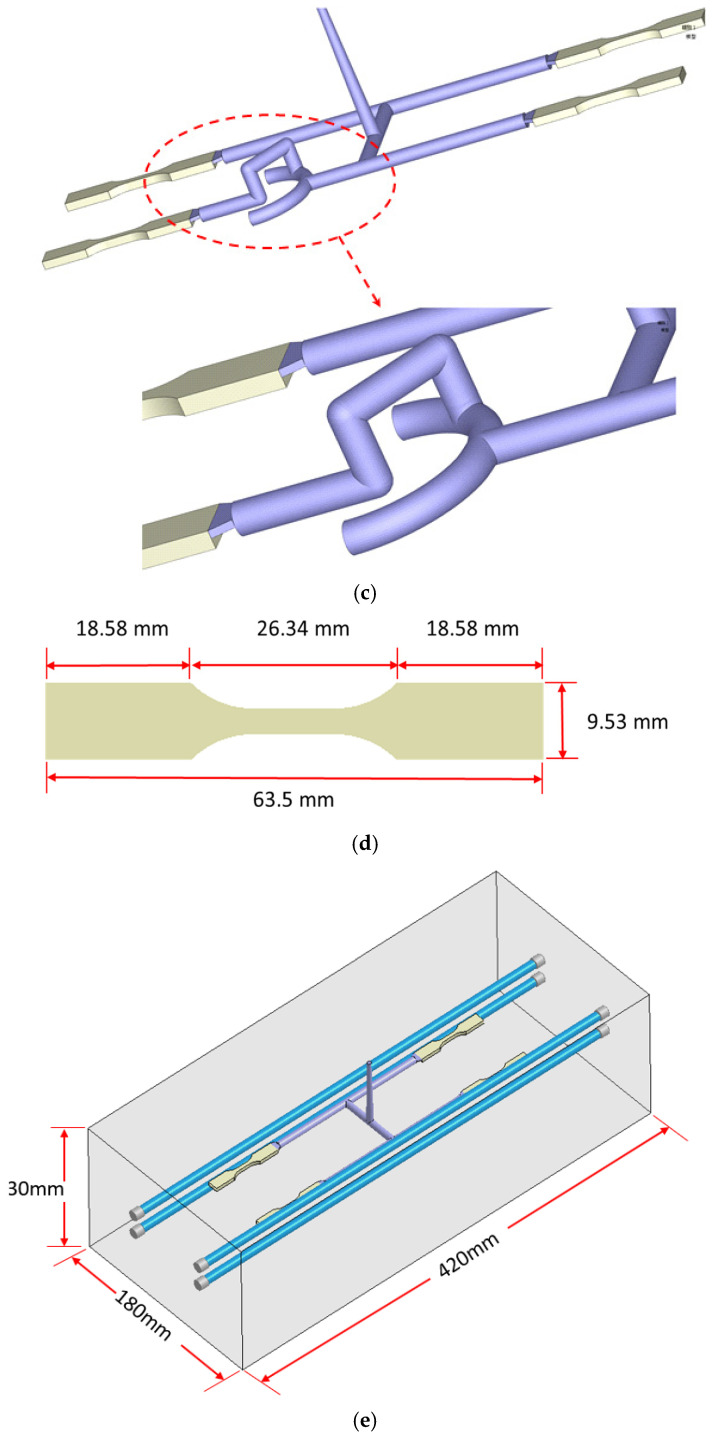
(**a**) Geometry and key dimensions of the balanced runner (BR) system; (**b**) geometry and key dimensions of the non-balanced runner (NBR) system; (**c**) the schematic diagram of the melt-rotation structure; (**d**) the dimension of products from 1A to 1D; (**e**) mold base configuration highlighting the cooling channel layout.

**Figure 3:**
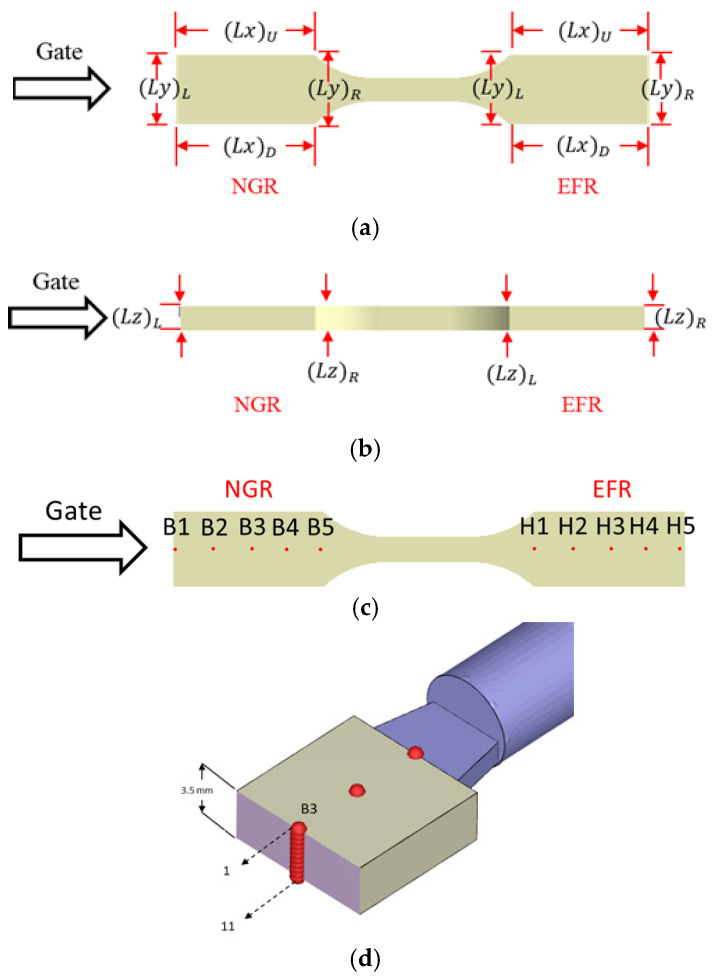
(**a**) Definition of specimen edge lengths in the flow (*x*) direction: upper (Lx)_U_ and lower (Lx)_D_; in the transverse (*y*) direction: left (Ly)_L_ and right (Ly)_R_; (**b**) definition of edge lengths in the thickness (*z*) direction: left (Lz)_L_ and right (Lz)_R_; (**c**) arrangement of the five fiber orientation measurement grids (B1–B5 and H1–H5) within each region; (**d**) layout of the eleven sensor nodes distributed through the thickness at every grid location.

In this study, Moldex3D 2022 version supplied by CoreTech System Co., Ltd, Hsinchu County, Taiwan is utilized as the CAE simulation. The product with some runner geometry models and related dimensions is shown in Figure 2, where Figure 2a is a one-by-four cavity with a balanced runner (BR) system. Figure 2b is a one-by-four cavity with a non-balanced runner (NBR) system. The diameters of the primary runner and the secondary runner are both 6 mm. Moreover, a melt-rotation runner structure is designed in the NBR system, which is set on the secondary runner of the 1C cavity. The geometric dimensions of the melt-rotation structure include a length of 20 mm and a height of 10 mm, as shown in Figure 2b. The schematic diagram of the melt-rotation structure is displayed in Figure 2c. It is mainly used to regulate the flow behavior of the melt in the unbalanced runner. In addition, each system contains an ASTM D638 Type V standard specimen. The dimension of the specimens is 63.5 mm × 9.53 mm × 3.5 mm, as shown in Figure 2d. Figure 2e displays the layout of the mold base and cooling channels. In addition, to investigate variations in the geometrical (macro-property variations) and the fiber orientation changes (micro-feature changes), specific locations were chosen for observation. Specifically, for each standard specimen, two zones are delineated according to the local flow conditions: the NGR and the EFR. To accommodate potential changes in the macroscopic geometry of the molded parts, we label the specimen’s edges as follows in Figure 3a: along the flow direction (*x*-axis), the upper and lower edges are denoted (Lx)_U_ and (Lx)_D_, respectively; perpendicular to the flow direction (*y*-axis), the left and right edges are designated (Ly)_L_ and (Ly)_R_. Likewise, the thickness direction (*z*-axis) is bounded by the left face, (Lz)_L_, and the right face, (Lz)_R_, as shown in Figure 3b. To resolve spatial variations in fiber orientation, each region is instrumented with five measurement grids (Figure 3c) designated B1–B5 and H1–H5. Every grid comprises eleven discrete measuring nodes arranged as illustrated in Figure 3d. Within the orientation tensors, the principal components are assigned as follows: A_11_ quantifies fiber alignment along the flow (x) direction, A_22_ captures alignment transverse to the flow (*y*) direction, and A_33_ represents alignment through the thickness (*z*) direction. Furthermore, the materials used are polypropylene (PP) and polypropylene containing 30 wt% glass fiber (30SFPP). The brand names of those materials are Globalene ST868M and SF7351, respectively, and are provided by LCY Chemical (Kaohsiung City, Taiwan). Injection molding was conducted under the following processing parameters: a filling time of 0.14 s, a packing time of 5 s with a packing pressure of 800 bar, and a cooling time of 30 s. The melt temperature was maintained at 220 °C, while the mold temperature was held at 25 °C. Moreover, to account for the flow-fiber coupling effect, during injection molding, the iARD-RPR model is used with parameters: C_I_ of 0.005; C_M_ of 0; α of 0.7. Additionally, the revised IISO model is applied to study the flow-fiber coupling effect, with parameters: $R_{T}^{0}\;$ of 20; ${\dot{\gamma }}_{C}$ of 10 [33,43].

### 3.2. Experimental System and Related Information

**Figure 4:**
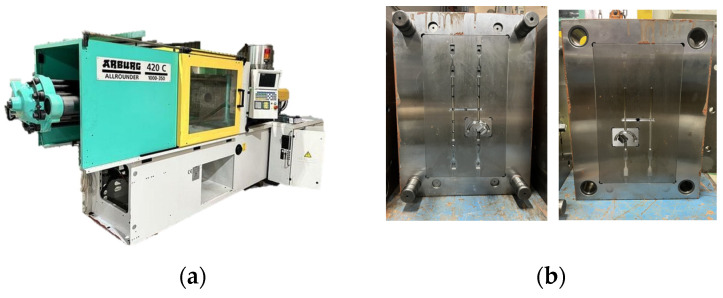
(**a**) The schematic of Arburg 420 C 1000-350-40 injection-molding machine; (**b**) detailed view of the mold cavity structure.

An Arburg 420 C 1000-350-40 injection-molding machine (Arburg, Warsaw, Poland) was employed for the experimental trials in Figure 4a; the corresponding mold assembly is shown in Figure 4b. Both the materials and the processing parameters were identical to those used in the simulation study. To quantify dimensional changes in the molded parts, the edge lengths defined in Figure 3a,b were measured on each specimen with a precision vernier caliper. To verify dimensional accuracy and representativeness, three molded specimens were randomly selected and measured; mean values and associated standard errors were then computed. Because the part geometry is uncomplicated and measurements were taken with a high-precision digital vernier caliper (resolution about 0.01 mm), the resulting uncertainty is negligible.

## 4. Results and Discussion

### 4.1. Exploration of Flow Behavior Variation Due to Melt-Rotation Effect

**Figure 5:**
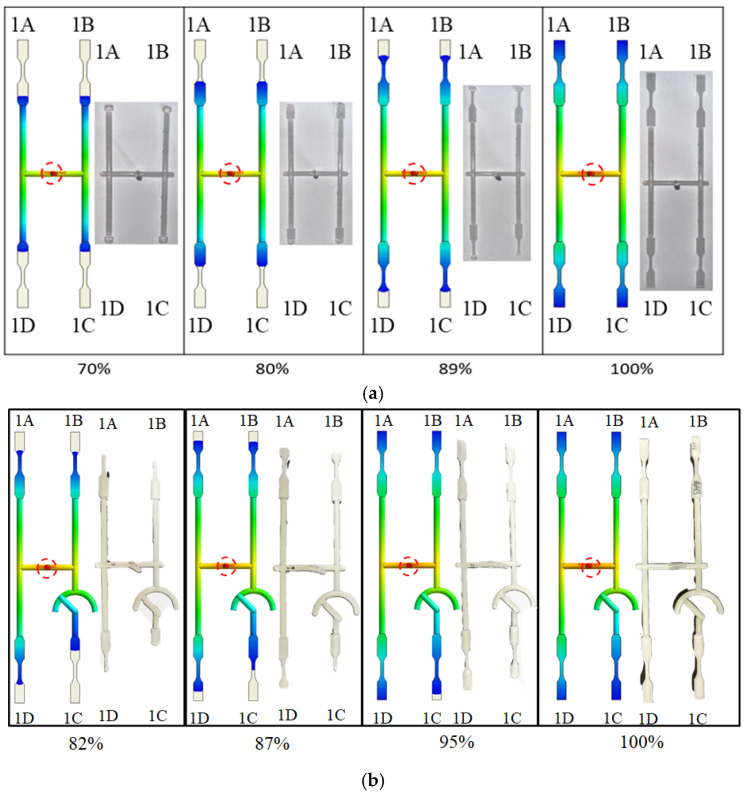
Comparison of melt front progression obtained from CAE simulation (left side) and short-shot experiments (right side): (**a**) BR system at 70–100% volumetric fill; (**b**) NBR system at 82–100% volumetric fill. The melt entrance is highlighted within the red circle in the figure. The colors in the figure represent the melt front time, ranging from short (red) to long (blue).

To evaluate how melt rotation alters flow behavior, a melt-rotation structure design was installed at the 1C station of the NBR system as displayed in Figure 2b. The first phase of the investigation examined the advancing melt front during filling by means of a melt-flow short-shot experiment, as detailed below. Figure 5a juxtaposes CAE predictions with short-shot observations for the BR system. At 70% fill, the simulation (left) shows the melt front in cavity 1C reaching the NGR, with the remaining three cavities exhibiting the same balanced progression; the experiment (right) reproduces this behavior. As filling increases to 80–89%, both simulation and experiment demonstrate a smooth advance of the melt front toward the EFR in every cavity. At 100% fill, the melt completes all four cavities uniformly in both datasets. This close agreement confirms that the BR’s symmetric geometry delivers a well-balanced flow field across the mold. Figure 5b illustrates the NBR system. At a filling volume of 82%, the CAE prediction (left) shows that the melt front in cavity 1C reaches only the near-gate region, whereas in the other three cavities it has already advanced to the mid-neck zone. This is clear evidence of flow imbalance. The short-shot photograph (right) confirms the same lag in cavity 1C. As the fill level rises from 87% to 95%, the simulated and experimental melt fronts remain in close agreement across the NBR system. The melt-rotation structure therefore retards the flow in cavity 1C, producing the observed unbalance; the resulting effects on part dimensions and fiber orientation evolution are examined in a subsequent section.

### 4.2. Discovery of the Geometrical Shrinkage Due to Melt-Rotation Effect

To quantify the influence of melt rotation on part shrinkage, the edge nomenclature illustrated in Figure 3a is adopted. In both the NGR and the EFR, the edges parallel to the flow (*x*) direction are labeled (Lx)_U_ (upper) and (Lx)_D_ (lower); the edges transverse to the flow (*y*) direction are labeled (Ly)_L_ and (Ly)_R_; and the edges along the thickness (*z*) direction are labeled (Lz)_L_ and (Lz)_R_. Moreover, the melt-rotation effect is evaluated by comparing the shrinkage of each of these individual edge lengths after molding. Furthermore, to quantify the influence of melt rotation on dimensional stability, the linear deviation of each edge is defined as

(17) $$|{\left|(L_{k})|\right|}_{j}={\left(L_{k}\right)}_{j}-{\left(L_{k}\right)}_{j,design}$$

where k ∈ {x, y, z} denotes the Cartesian direction and the subscript j specifies the particular edge: U (upper) or D (lower) in the x-direction, L (left) or R (right) in the y-direction, and likewise L or R in the *z*-direction. The “design” refers to the nominal dimension taken from the cavity model. Here ${\left(L_{x}\right)}_{U,\;design}$ and ${\left(L_{x}\right)}_{D,\;design}$ are 18.58 mm; ${\left(L_{y}\right)}_{L,\;design}$ and ${\left(L_{y}\right)}_{R,\;design}$ are 9.53 mm; and ${\left(L_{z}\right)}_{L,\;design}$ and ${\left(L_{z}\right)}_{R,\;design}$ are 3.5 mm, respectively. A value of zero for $|{\left|(L_{k})|\right|}_{j}$ indicates that the corresponding edge length deviation remains unchanged relative to the nominal design, exhibiting neither shrinkage nor expansion.

**Figure 6:**
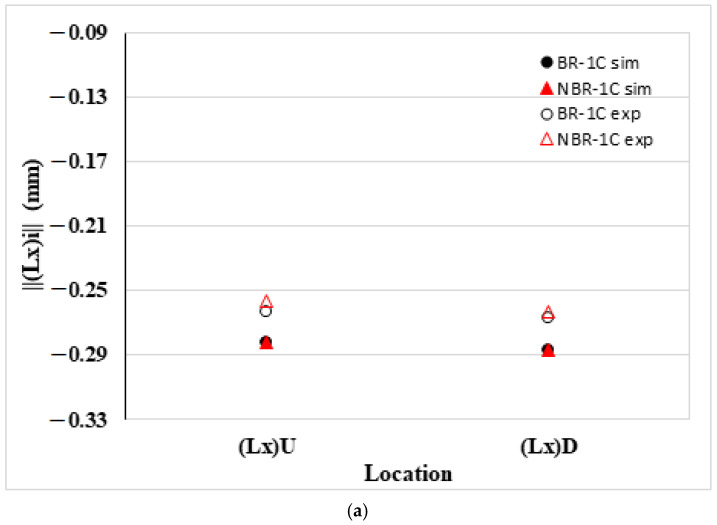

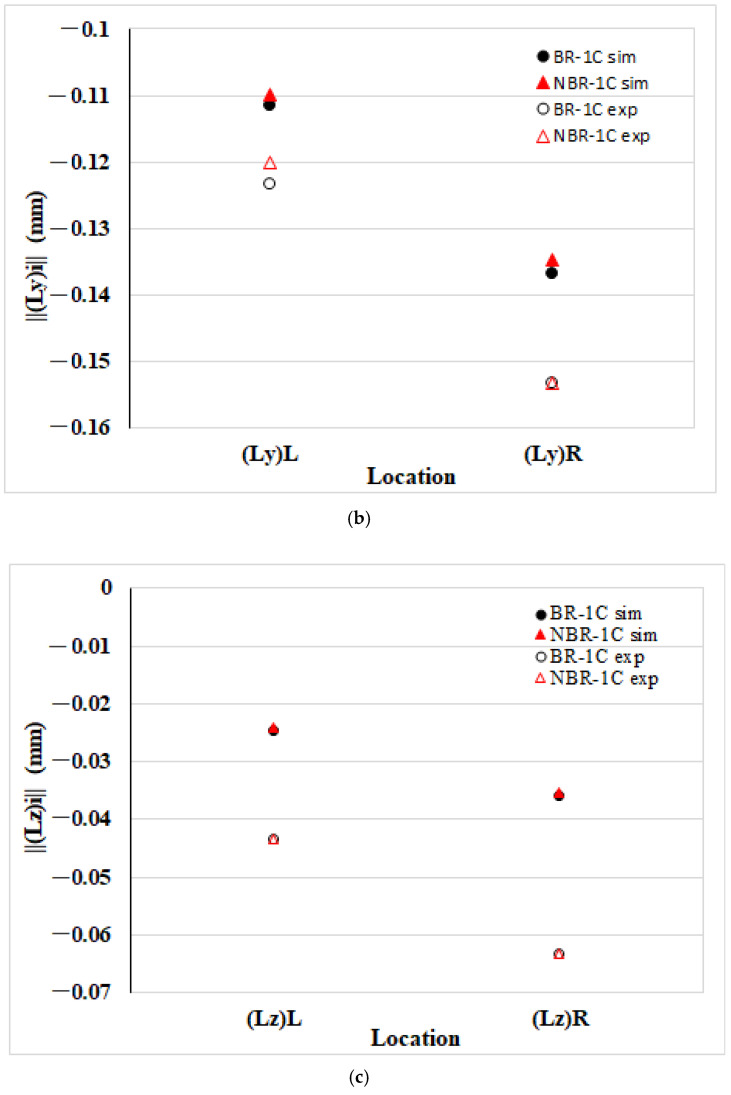
Comparison of the edge length deviation between simulated and measured dimensions of the PP specimen in the NGR: (**a**) flow direction length deviation $|{\left|(L_{x})|\right|}_{U}$ and $|{\left|(L_{x})|\right|}_{D};$ (**b**) transverse (cross-flow) length deviation $|{\left|(L_{y})|\right|}_{L}$ and $|{\left|(L_{y})|\right|}_{R}$; and (**c**) length deviation in thickness direction and thickness $|{\left|(L_{z})|\right|}_{L}$ and $|{\left|(L_{z})|\right|}_{R}$.

Figure 6 compares the edge-wise dimensional changes of the PP specimen in the NGR after melt rotation. In Figure 6a, the flow direction that the CAE model predicts is only a marginal contraction of both the upper and lower edges, $|{\left|(L_{x})|\right|}_{U}$ and $|{\left|(L_{x})|\right|}_{D}$; the experimental data show an even smaller shrinkage, confirming that melt rotation has little effect on these dimensions. In the transverse direction, an asymmetry emerges as shown in Figure 6b: the edge nearest to the gate, $|{\left|(L_{y})|\right|}_{L}$, contracts less than the opposite edge, $|{\left|(L_{y})|\right|}_{R}$, because the former benefits from the packing pressure. Melt rotation also reduces the overall transverse shrinkage of both $|{\left|(L_{y})|\right|}_{L}$ and $|{\left|(L_{y})|\right|}_{R}$ compared with the BR baseline. In Figure 6c, the thickness direction data show negligible differences between simulation and experiment. Overall, the experimental measurements closely follow the simulation trends, validating the accuracy of the model.

**Figure 7:**
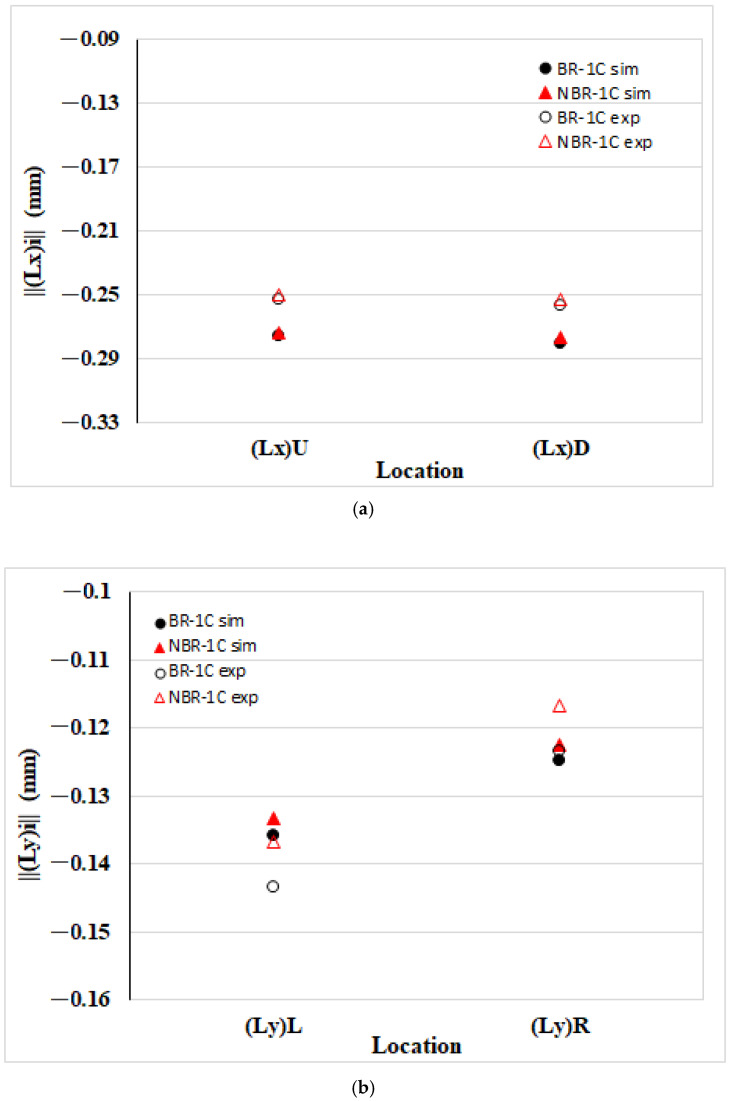

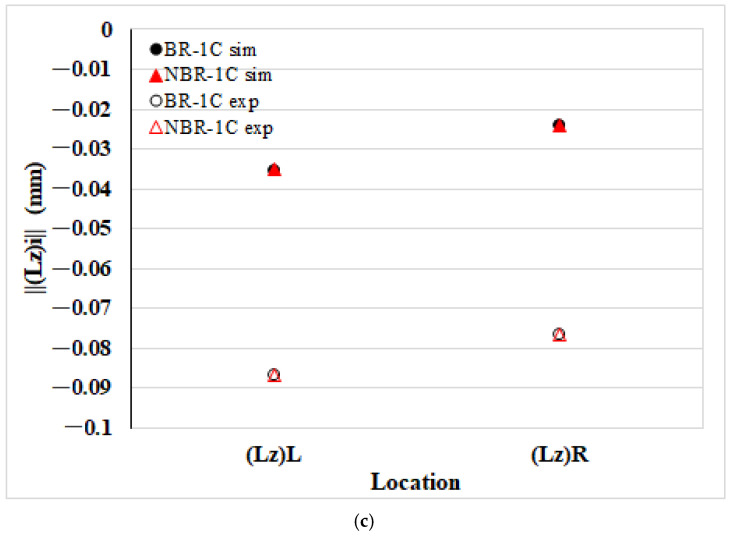
Comparison of the edge length deviation between simulated and measured dimensions of the PP specimen in the EFR: (**a**) flow direction length deviation $|{\left|(L_{x})|\right|}_{U}$ and $|{\left|(L_{x})|\right|}_{D}$; (**b**) transverse (cross-flow) length deviation $|{\left|(L_{y})|\right|}_{L}$ and $|{\left|(L_{y})|\right|}_{R}$; (**c**) length deviation in thickness direction and thickness $|{\left|(L_{z})|\right|}_{L}$ and $|{\left|(L_{z})|\right|}_{R}$.

Figure 7 presents the edge length variations of PP parts in the EFR after the melt rotation. In Figure 7a, both the simulation and the experiment show that the flow direction edges, $|{\left|(L_{x})|\right|}_{U}$ and $|{\left|(L_{x})|\right|}_{D}$, contract after the melt rotated, and the trends agree closely. Figure 7b depicts the transverse dimensions: here, unlike in the near-gate region, the upstream edge $|{\left|(L_{y})|\right|}_{L}$ shrinks more than the downstream edge $|{\left|(L_{y})|\right|}_{R}$. This may be due to the original fluid moving at high speed in the flow direction. As it approached the filling end boundary, inertia caused the melt to disperse in the vertical and thickness directions. It results in the downstream zone alleviating transverse contraction, leading to smaller overall deformation there. After the melt rotated, transverse shrinkage in the NBR system decreased markedly at both $|{\left|(L_{y})|\right|}_{L}$ and $|{\left|(L_{y})|\right|}_{R}$; experimental measurements confirmed the trend by numerical prediction. Figure 7c illustrates the thickness direction response: the upstream edge $|{\left|(L_{z})|\right|}_{L}$ contracts more than the downstream edge $|{\left|(L_{y})|\right|}_{R}$. Relative to the balanced runner baseline, melt rotating exerts only a minor additional influence on $|{\left|(L_{z})|\right|}_{L}$ and $|{\left|(L_{y})|\right|}_{R}$. Once again, simulation results are in excellent agreement with the experimental data.

Overall, the simulated edge shrinkage predictions for PP specimens both with and without melt rotation in the NGR and EFR regions closely match the experimental measurements.

**Figure 8:**
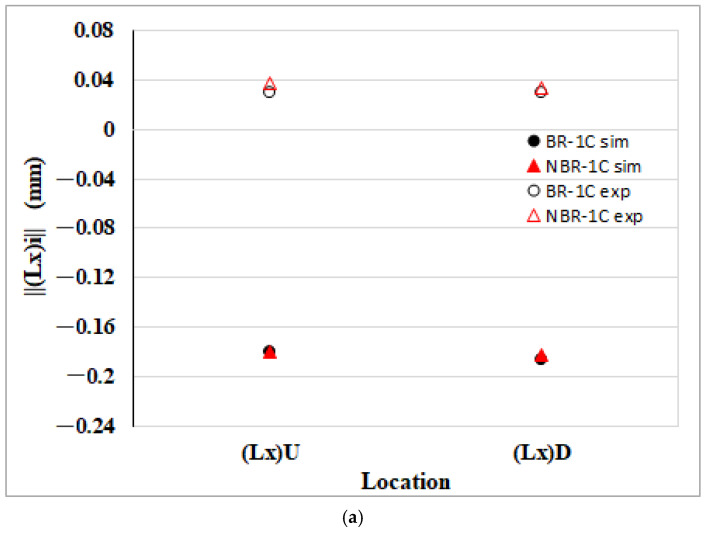

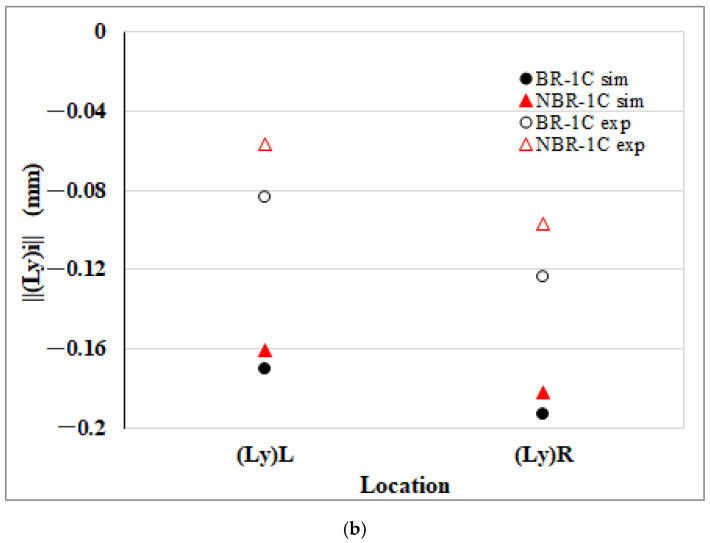

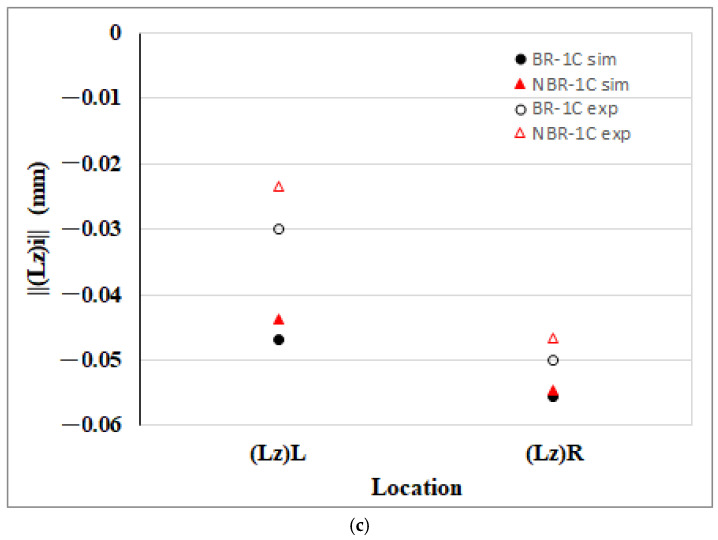
Comparison of the edge length deviation between simulated and measured dimensions of the 30SFPP specimen in the NGR: (**a**) flow direction length deviation $|{\left|(L_{x})|\right|}_{U}$ and $|{\left|(L_{x})|\right|}_{D};$ (**b**) transverse (cross-flow) length deviation $|{\left|(L_{y})|\right|}_{L}$ and $|{\left|(L_{y})|\right|}_{R}$; and (**c**) length deviation in thickness direction $|{\left|(L_{z})|\right|}_{L}$ and $|{\left|(L_{z})|\right|}_{R}$.

Figure 8 compares edge length variations in the NGR of 30 wt% glass fiber PP (30SFPP) parts with and without melt rotating. In Figure 8a, the CAE model predicts a slight reduction in the flow direction dimensions, $|{\left|(L_{x})|\right|}_{U}$ and $|{\left|(L_{x})|\right|}_{D}$, after the melt rotated. The experimental data show less contraction of the same edges, yet the overall trend agrees with the simulation. Figure 8b displays the transverse (cross-flow) dimensional changes predicted after the melt rotated. As in the PP system, the upstream edge $|{\left|(L_{y})|\right|}_{L}$ (NGR) contracts less than the downstream edge $|{\left|(L_{y})|\right|}_{R}$, most likely because the upstream region experiences more effective packing pressure. Moreover, relative to the BR baseline, both $|{\left|(L_{y})|\right|}_{L}$ and $|{\left|(L_{y})|\right|}_{R}$ exhibit markedly lower shrinkage in the NBR configuration, demonstrating that melt rotating mitigates transverse shrinkage in the NGR zone. The experimental data follow the same trend, corroborating the simulation. Figure 8c compares simulation and experimental results for the thickness direction. After melt rotating, shrinkage along both $|{\left|(L_{z})|\right|}_{L}$ and $|{\left|(L_{z})|\right|}_{R}$ decreases markedly, mirroring the behavior observed in the transverse direction. The upstream edge $|{\left|(L_{z})|\right|}_{L}$ (near the gate) contracts less than the downstream edge $|{\left|(L_{y})|\right|}_{R}$, again reflecting the more effective packing pressure at the gate. Overall, the numerical predictions align closely with the measurements, confirming the model’s ability to capture dimensional shrinkage in the NGR region.

**Figure 9:**
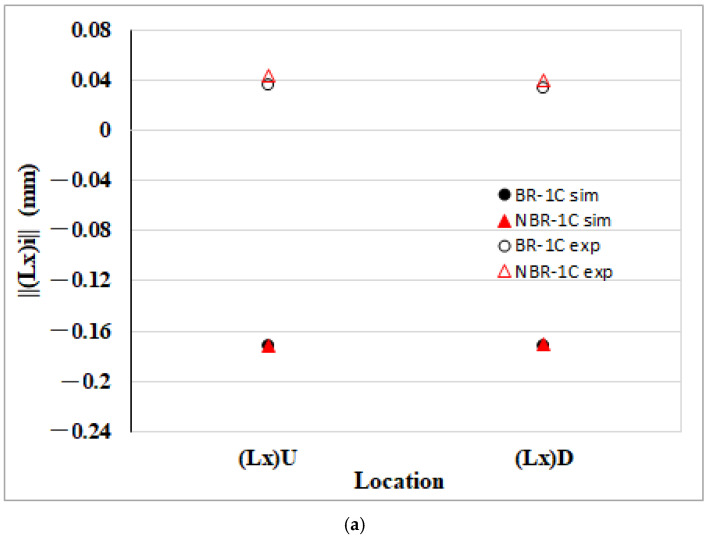

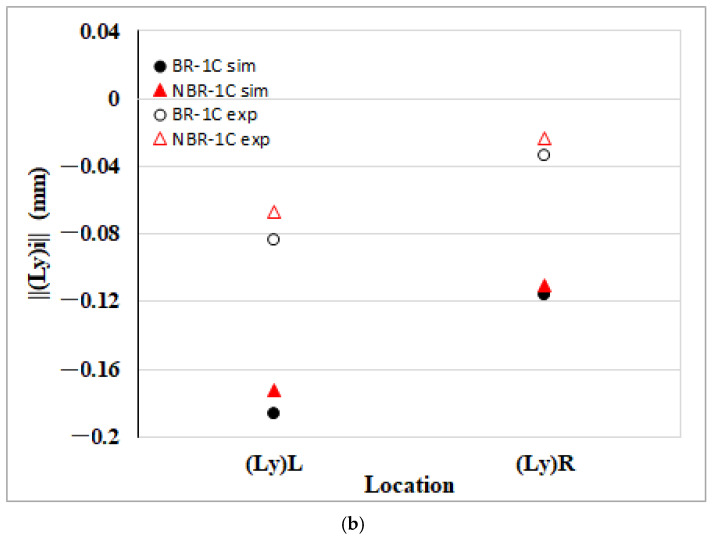

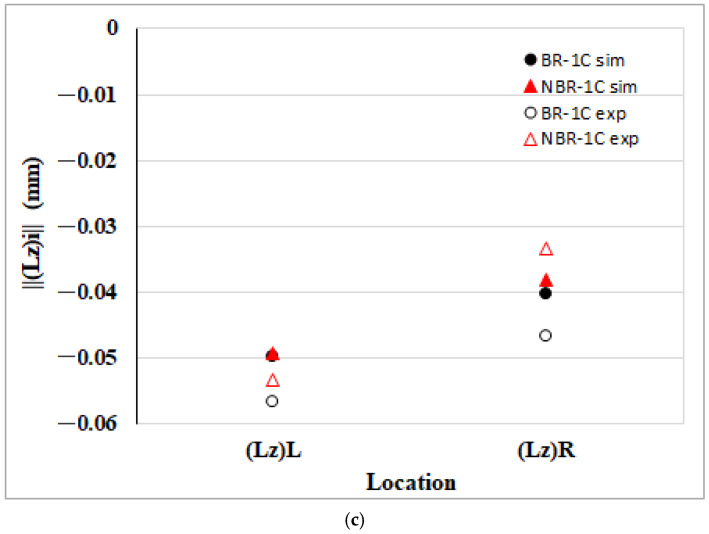
Comparison of the edge length deviation between simulated and measured dimensions of the 30SFPP specimen in the EFR: (**a**) flow direction length deviation $|{\left|(L_{x})|\right|}_{U}$ and $|{\left|(L_{x})|\right|}_{D}$; (**b**) transverse (cross-flow) length deviation $|{\left|(L_{y})|\right|}_{L}$ and $|{\left|(L_{y})|\right|}_{R}$; (**c**) length deviation in thickness direction and thickness $|{\left|(L_{z})|\right|}_{L}$ and $|{\left|(L_{z})|\right|}_{R}$.

Figure 9 summarizes the edge length variations of the 30SFPP specimens in the EFR region with and without melt rotation. As shown in Figure 9a, the CAE model predicts only a slight contraction of the flow direction edges, $|{\left|(L_{x})|\right|}_{U}$ and $|{\left|(L_{x})|\right|}_{D}$, after the melt is rotated, whereas the experimental data reveal a pronounced shrinkage of both edges. Figure 9b further indicates an asymmetric transverse response: the upstream edge $|{\left|(L_{y})|\right|}_{L}$ shrinks more than the downstream edge $|{\left|(L_{y})|\right|}_{R}$. This disparity is attributed to flow–fiber coupling, whereby interaction between the polymer melt and the short glass fibers promotes alignment perpendicular to the flow, locally increasing stiffness and thereby mitigating transverse deformation in the downstream zone. Comparison of the BR and NBR configurations reveals that melt rotation markedly reduces transverse shrinkage: both the upstream edge $|{\left|(L_{y})|\right|}_{L}$ and the downstream edge $|{\left|(L_{y})|\right|}_{R}$ contract far less in the NBR system. The CAE predictions capture this reduction accurately, and the experimental measurements corroborate the simulated trend, confirming the reliability of the numerical model. Figure 9c compares the thickness direction shrinkage derived from simulation and experiment. The numerical results indicate that the upstream edge, $|{\left|(L_{z})|\right|}_{L}$, contracts more than the downstream edge, $|{\left|(L_{z})|\right|}_{R}$; this behavior is attributed to flow–fiber coupling, which promotes fiber alignment through the thickness and thereby suppresses shrinkage at the downstream location. When the melt rotating structure is introduced (NBR configuration), both $|{\left|(L_{z})|\right|}_{L}$ and $|{\left|(L_{z})|\right|}_{R}$ exhibit substantially lower contraction than in the BR baseline, demonstrating that the melt rotation is able to alleviate thickness shrinkage. The experimental data closely follow the simulated trends, further validating the predictive accuracy of the CAE model.

Moreover, upon examining Figure 9b, we observe that the flow-fiber coupling effect of 30SFPP in the EFR region of the injected parts causes a significant upstream contraction in the cross-flow direction, resulting in a left-right asymmetry. Comparing with recent literature [38], it is noted that despite major changes in the product and flow channel design, the flow-fiber coupling effect at the end of the flow field remains consistent. Additionally, a comparison of Figure 7b and Figure 9b shows that changing the material from PP to 30SFPP increases the difference in length contraction of $|{\left|(L_{z})|\right|}_{L}$ and $|{\left|(L_{z})|\right|}_{R}$ from 0.011 mm in the PP system to 0.07 mm in the 30SFPP system. This demonstrates the significant and specific influence of the flow-fiber coupling effect.

Overall, the CAE predictions of edge-wise shrinkage for the 30 wt% glass fiber-reinforced PP parts, evaluated in both the NGR and EFR regions, with and without melt rotation, agree closely with the experimental measurements.

### 4.3. Discovery of Fiber Orientation Changes Due to Melt Rotation

**Figure 10:**
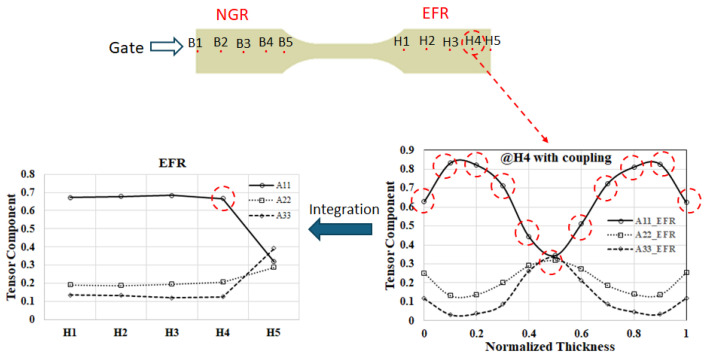
Schematic of the averaged fiber orientation tensor for each sampling site extracted as a function of position along the flow path. The red-circled value on the left represents the integral sum of the values at the eleven positions shown on the right.

To assess how melt rotation alters fiber orientation in FRP moldings, we carried out a detailed CAE study. In both the NGR and EFR regions, eleven sampling nodes were positioned through the thickness at each of the ten measurement sites (B1–B5 and H1–H5; see Figure 3b). For every node, the fiber orientation tensor (A_11_, A_22_, A_33_) was calculated and stored. To compare spatial variations, the tensors obtained at the eleven nodes of a given site were averaged to produce a representative orientation tensor for that location; these averaged results are summarized in Figure 10.

**Figure 11:**
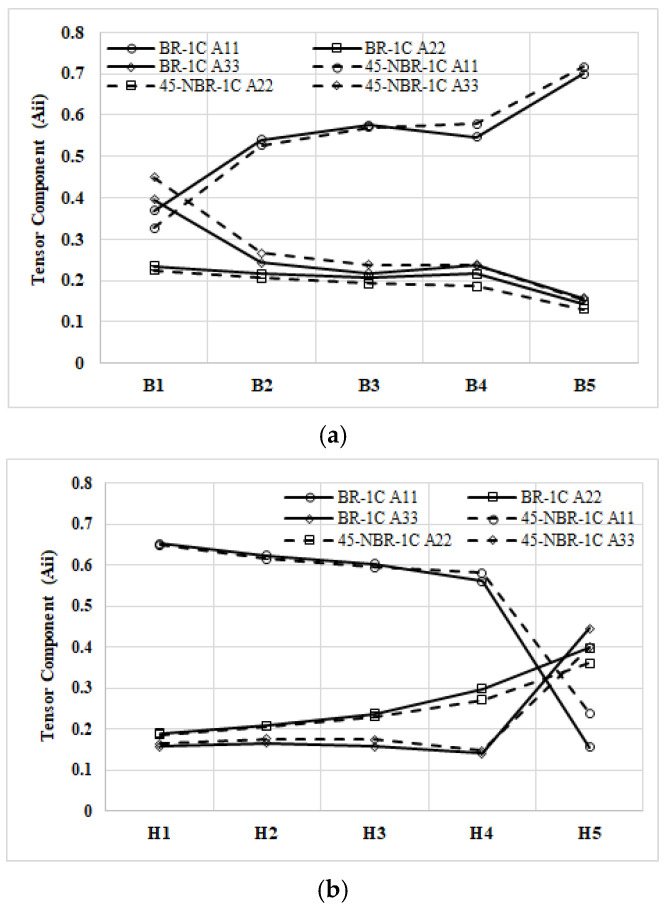
Variation of the averaged fiber orientation tensor along the flow path under melt rotation: (**a**) at NGR; (**b**) at EFR.

Figure 11a compares the fiber orientation tensors of the BR and NBR systems in the NGR region. When the melt is rotated, the fiber orientation tensor in the flow direction (A_11_) initially declines from the upstream position B1 and rises again toward the downstream position B5. Conversely, the fiber orientation tensor in the cross-flow direction (A_22_) diminishes steadily along the same path, while the fiber orientation tensor in the thickness component (A_33_) shows the opposite trend, that is, increasing from B1 and then decreasing toward B5. Figure 11b illustrates the evolution of the fiber orientation tensor in the EFR region for both the BR and NBR systems. Under melt rotation, the fiber orientation tensor in the flow direction (A_11_) first decreases from the upstream position H1 and then rises toward the downstream position H5. The transverse component (A_22_) steadily diminishes along the same path. By contrast, the fiber orientation tensor in the thickness component (A_33_) shows the opposite behavior, increasing from H1 to mid-cavity before declining toward H5.

These findings indicate that melt rotation induces measurable, yet moderate, alterations in fiber orientation within the injection-molded FRP parts. The extent to which these microstructural adjustments translate into macroscopic dimensional responses, however, remains unclear and warrants deeper investigation.

### 4.4. Discovery of the Correlation Between Geometrical Variation and Fiber Orientation Changes Due to Melt-Rotation Effect

To elucidate the link between macroscopic dimensional shrinkage and microscopic fiber orientation changes after melt rotation, we isolated the incremental effect of the melt rotation. Building on the edge length shrinkage data in Section 4.2, where simulated trends closely matched the measurements in both the NGR and EFR regions, we introduce a “net shrinkage change” that quantifies the sole contribution of melt rotation. The net change in edge shrinkage attributable to melt rotation is defined by

(18) $${\Delta (L_{k})}_{j}={\left(L_{k}\right)}_{j,NBR}-{\left(L_{k}\right)}_{j,BR}$$

where k ∈ {x, y, z} denotes the Cartesian direction and the subscript j specifies the particular edge: U (upper) or D (lower) in the *x*-direction, L (left) or R (right) in the *y*-direction, and likewise L or R in the *z*-direction. Here, $\;{\left(L_{k}\right)}_{j,NBR}$ is the measured edge length obtained with the NBR configuration, i.e., with melt rotation, whereas ${\left(L_{k}\right)}_{j,BR}$ is the corresponding length for the BR baseline.

Similarly, to quantify the incremental influence of melt rotation on fiber orientation, we define the net orientation change, Δ*Aii*, as

(19) $$\Delta A_{ii}={\left(A_{ii}\right)}_{NBR}-{\left(A_{ii}\right)}_{BR}$$

where *i* can be 1, 2, and 3 that correspond to the Cartesian directions *x*-, *y*-, and *z*, respectively; ${\left(A_{ii}\right)}_{NBR}$ is the component obtained with the NBR configuration, i.e., with melt rotation, whereas ${\left(A_{ii}\right)}_{BR}$ is the baseline value measured in the BR system.

**Figure 12:**
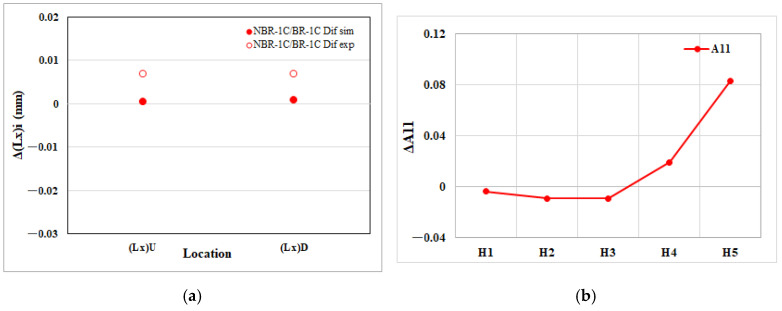
Melt rotating induced changes in the EFR region of the injection-molded part: (**a**) net effect on flow direction edge length shrinkage (Lx); (**b**) net effect on the flow direction fiber orientation tensor component (A_11_).

Figure 12a depicts the net change in the flow direction edge length within the EFR region attributable to melt rotation. The CAE simulation shows that both the upper and lower flow direction edges, ${\Delta (L_{x})}_{U}$ and ${\Delta (L_{x})}_{D}$, increase in size relative to the balanced runner baseline, indicating a reduction in longitudinal shrinkage. The experimental measurements confirm this enlargement, demonstrating good agreement with the numerical prediction and validating the melt rotation’s ability to alleviate flow direction contraction at the end of fill. On the other hand, Figure 12b displays the net change in the flow parallel fiber orientation component (A_11_) within the EFR region. As the melt progresses from the upstream position H1 to the downstream position H5, ΔA_11_ rises steadily, indicating a progressively stronger alignment of fibers along the flow direction. This microstructural realignment provides the internal driving force behind the enlarged flow direction edges observed in Figure 12a, demonstrating a clear correspondence between the microscopic orientation shift and the macroscopic reduction in longitudinal shrinkage.

**Figure 13:**
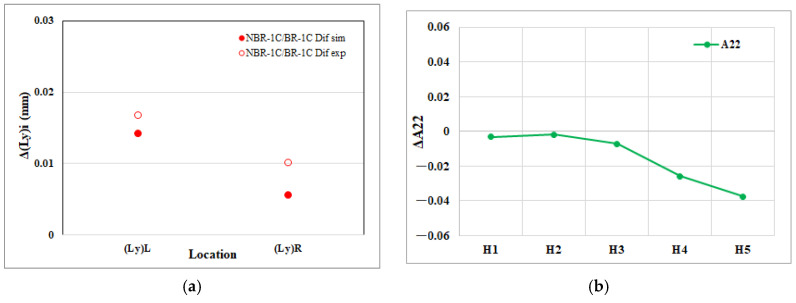
Melt rotating induced changes in the EFR region of the injection-molded part: (**a**) net effect on cross-flow direction edge length shrinkage (Ly); (**b**) net effect on the cross-flow direction fiber orientation tensor component (A_22_).

Figure 13a illustrates the net change in transverse edge length within the EFR region that arises from melt rotation. The CAE results indicate a noticeably larger expansion (i.e., more positive ΔL) for the upstream transverse edge, ${\Delta (L_{y})}_{L}$ than for the downstream edge, ${\Delta (L_{y})}_{R}$. The experimental measurements reproduce this disparity almost exactly, confirming the numerical prediction and underscoring the reliability of the simulation in capturing melt-rotation-induced anisotropic shrinkage. Figure 13b presents the net change in the transverse fiber orientation component (A_22_) across the EFR region. As the flow proceeds downstream from H1 to H5, ΔA_22_ diminishes progressively, indicating a gradual loss of fiber alignment perpendicular to the flow. This reduction in transverse orientation corresponds to the larger expansion (i.e., smaller shrinkage) observed on the upstream edge ${\Delta (L_{y})}_{L}$ relative to the downstream edge ${\Delta (L_{y})}_{R}$ in Figure 13a. The close agreement between the microstructural trend in (A_22_) and the macroscopic edge length response confirms that melt rotation controls transverse dimensional change primarily through its influence on fiber re-orientation.

**Figure 14:**
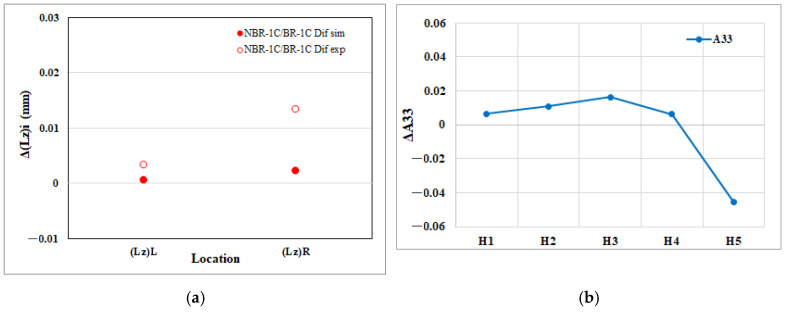
Melt rotating induced changes in the EFR region of the injection-molded part: (**a**) net effect on thickness direction edge length shrinkage (Lz); (**b**) net effect on the thickness direction fiber orientation tensor component (A_33_).

Figure 14a depicts the net shrinkage in thickness for the EFR region attributable to melt rotation. The CAE model predicts that the upstream thickness edge, ${\Delta (L_{z})}_{L}$, contracts more than the downstream edge, ${\Delta (L_{z})}_{R}$. The experimental measurements replicate this disparity, confirming both the magnitude and direction of the simulated trend and thereby validating the model’s ability to capture melt-rotation effects in the thickness dimension. Moreover, Figure 14b reveals how melt rotation alters the through-thickness fiber orientation component (A_33_) in the EFR region. As the flow progresses from the upstream position H1 toward H4, the net increase in (A_33_) becomes progressively larger, indicating that fibers are increasingly rotated out of the flow plane. Unexpectedly, between H4 and H5, this trend reverses and ΔA_33_ drops sharply. The rising A_33_ upstream explains why the thickness edge ${\Delta (L_{z})}_{L}$ contracts more than ${\Delta (L_{z})}_{R}$; however, the sudden decline in A_33_ at H5 breaks this correspondence, so the downstream thickness change is not fully mirrored by the fiber orientation. Hence, in contrast to the flow and transverse direction results, the macroscopic thickness shrinkage is only partly captured by the microscopic variation in A_33_, suggesting that additional factors such as local cooling rate or pressure decay also contribute to the final thickness dimension at the mold exit.

**Figure 15:**
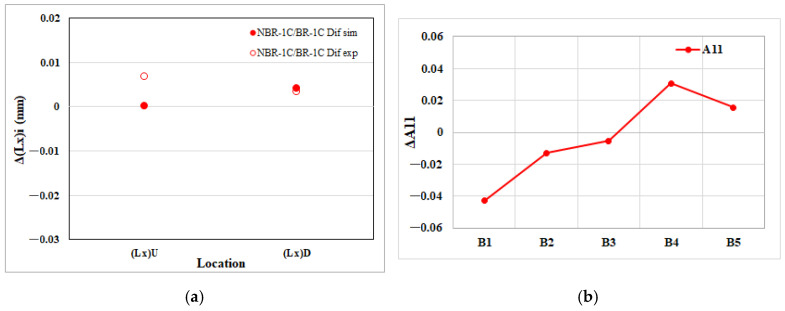
Melt rotating induced changes in the NGR region of the injection-molded part: (**a**) net effect on flow direction edge length shrinkage (Lx); (**b**) net effect on the flow direction fiber orientation tensor component (A_11_).

**Figure 16:**
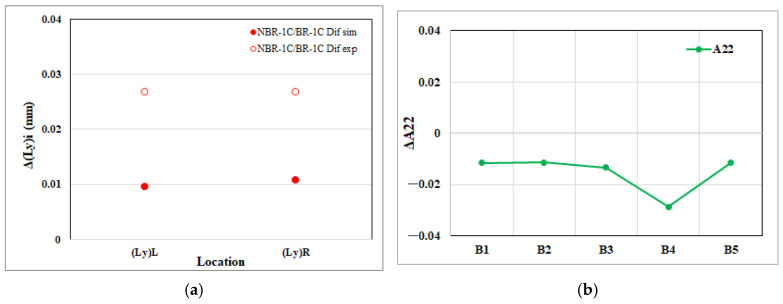
Melt rotating induced changes in the NGR region of the injection-molded part: (**a**) net effect on cross-flow direction edge length shrinkage (Ly); (**b**) net effect on the cross-flow direction fiber orientation tensor component (A_22_).

**Figure 17:**
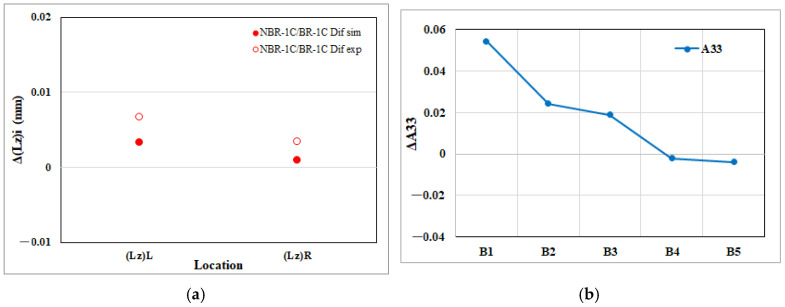
Melt rotating induced changes in the NGR region of the injection-molded part: (**a**) net effect on thickness direction edge length shrinkage (Lz); (**b**) net effect on the thickness direction fiber orientation tensor component (A_33_).

The melt-rotation effect produces broadly coherent responses at both length scales and fiber orientation. In the EFR region, the net variations in edge dimensions mirror the corresponding shifts in the fiber orientation tensor, indicating that microstructural re-orientation is the primary driver of macroscopic shrinkage and expansion. Furthermore, a detailed breakdown for the NGR region, presented in Figure 15, Figure 16 and Figure 17, confirms the same one-to-one correspondence: every change in an individual edge length is matched by a proportional change in the associated tensor component. These findings reinforce the conclusion that melt rotation influences part geometry predominantly through its effect on fiber alignment.

### 4.5. Mechanistic Discussion

**Figure 18:**
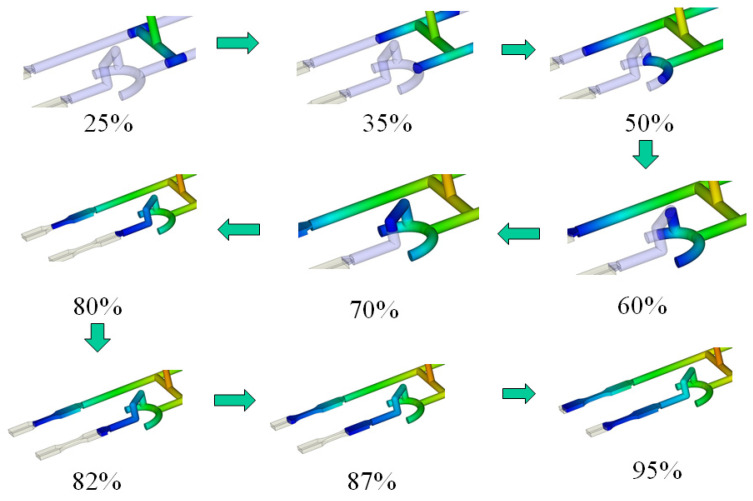
The schematic illustrates the polymer flow behavior through the melt rotation structure.

**Figure 19:**
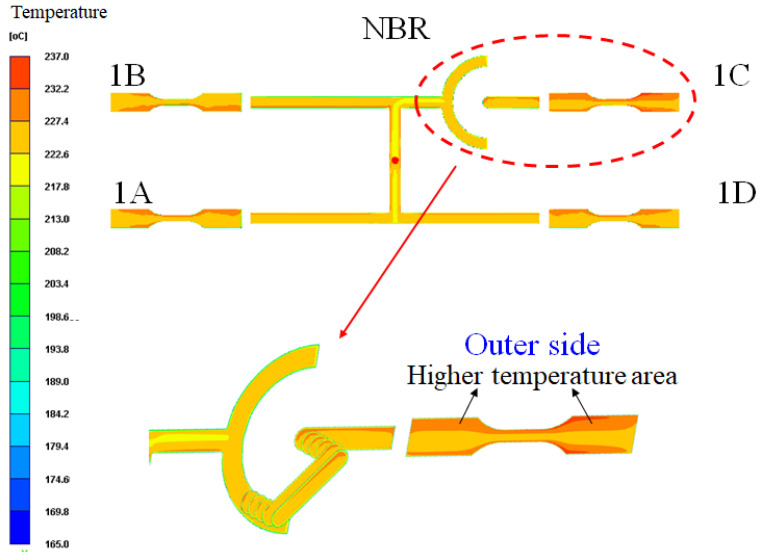
Melt-rotation effect on internal temperature distribution at the end of filling.

To investigate how the melt-rotation effect influences both the intrinsic fiber microstructure and the external geometric dimensions, CAE analysis was conducted. Figure 18 illustrates the case of FRP melt entering the melt-rotation structure of Cavity 1C. When the filling stage reaches approximately 25–35%, the melt flows sequentially from the sprue into the primary runner, then into the secondary runner, and subsequently toward the bottom of the melt-rotation structure. As filling progresses to around 50–70%, the melt gradually rises from the bottom of the melt-rotation structure into the upper runner of the inverted U-shaped configuration, and finally returns toward the gate, thereby entering the cavity and completing the filling process. At the end of filling, Figure 19 shows that the temperature distribution of the melt in Cavity 1C is markedly different from that in the other three cavities due to the melt-rotation effect. Specifically, after the melt is reorganized, a localized high-temperature region appears on the outer side of the 1C product. From the perspective of the melt-rotation structure, before the melt is fully overturned, the high-temperature melt accumulates near the outer region, ultimately leading to this distinctive temperature distribution. This principle—whereby melt rotation alters the internal temperature distribution and consequently changes flow behavior—is consistent with the findings reported in the literature [36,37,38] and aligns with the mechanism of flow-field improvement through melt rotation described in the literature [40].

**Figure 20:**
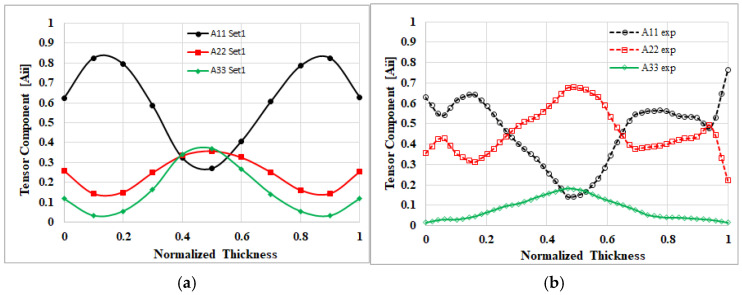
Comparison of fiber orientation results at the H3 location in this study: (**a**) simulation; (**b**) experimental observation.

Moreover, we investigated the correlation between the geometric shrinkage of the finished product and changes in internal fiber orientation. The correlation in the thickness direction within the EFR was found to be not entirely consistent. To further clarify this, a micro-CT scan analysis was conducted specifically for this region. The same experimental method and procedure as described in Ref. [44] were applied, and the simulation results were compared with the experimental FOD measurements, as shown in Figure 20. As illustrated, the overall trends of A_11_, A_22_, and A_33_ show that the simulation analysis and experimental results are consistent with those reported in Ref. [44], with the level of agreement falling within a reasonable range. However, the simulation results in this study indicate that the A_11_ tensor is overestimated, while the A_22_ tensor is underestimated, suggesting that the flow–fiber coupling effect in actual injection-molded products is considerably stronger than that captured by the simulation. According to Ref. [27], in more complex geometries, the initial dimensionless Trouton ratio parameter may need to be set as high as 375. In the present study, this parameter was only set to 20, which may explain the discrepancy between the simulation and experimental results, likely due to an underestimated flow–fiber coupling parameter.

## 5. Conclusions

We sincerely thank the reviewer for the valuable suggestions. The focus of this study is on the asymmetric shrinkage observed between the upstream and downstream regions of FRP injection-molded products, which arises from processing-induced effects. Under conditions where product and mold design remain unchanged, such asymmetric shrinkage cannot be effectively mitigated solely by adjusting conventional injection-molding operating parameters. To address this challenge, we developed a self-designed melt-rotation structure to alter the intrinsic characteristics of the melt within the flow channel. This approach enables modification of both the internal fiber orientation distribution (microscopic properties) and the external geometric shrinkage behavior (macroscopic properties) of the molded product. The main conclusions of this study can be summarized as follows:

(1) This research employed FRP materials to fabricate injection-molded parts utilizing a one-by-four multi-cavity mold with and without balanced runner systems. Through a combination of simulation analysis and experimental validation, it was observed that the products exhibited uneven shrinkage between the upstream and downstream edge sides at the end of the fill region. The findings identified the primary cause of this uneven shrinkage as the flow-fiber coupling effect, which persists even in the presence of complex flow channels and intricate geometric configurations.
(2) By implementing the melt-rotation design in the NBR system, the polymer melt-flow field is intentionally modified, thereby mitigating geometric shrinkage in the molded part. Specifically, compared with the original BR system, the NBR configuration substantially reduces the upstream-downstream asymmetry in transverse (Ly) shrinkage in the EFR region. The findings indicate that controlling the melt-rotation effect helps reduce asymmetric shrinkage in FRP injection-molded parts.
(3) When switching from the BR system to the NBR system, the melt-rotation effect was found to distinctly alter the fiber orientation distribution within the melt. Specifically, at location H5 of EFR, the fiber orientation tensor component A_11_ in the flow direction increased by 0.083, while A_22_ in the transverse direction decreased by 0.0376, and A_33_ in the thickness direction decreased by 0.0462. These results demonstrate that the melt-rotation effect can mitigate the influence of flow–fiber coupling.
(4) Switching from the BR to the NBR system showed that the net changes in FOD corresponded directly with the net changes in shrinkage behavior. In the EFR, ΔA_22_ decreased by 0.0376, improving upstream/downstream shrinkage asymmetry in the cross-flow direction (Ly) by about 0.1%.
(5) The results further indicate that geometric precision in future FRP injection-molded products can be achieved by tailoring the flow field to guide internal fiber orientation, thereby enabling tighter dimensional tolerances. This approach is particularly valuable for developing lightweight FRP products with stringent precision requirements.
(6) Future work will investigate alternative melt-rotation designs and the optimization of model-internal parameters in FOD prediction.

## Data Availability

The data presented in this study are available on request from the corresponding author.

## References

[1] (2025). Composites World Report. Composites End Markets: Automotive. https://www.compositesworld.com/articles/composites-end-markets-automotive-2025.

[2] Othman R., Ismail N.I., Pahmi M.A.A.H., Basri M.H.M., Sharudin H., Hemdi A.R. (2018). Application of carbon fiber reinforced plastics in automotive industry: A review. J. Mech. Manuf..

[3] Der A., Kaluza A., Reimer L., Herrmann C., Thiede S. (2022). Integration of energy oriented manufacturing simulation into the life cycle evaluation of lightweight body parts. Int. J. Precis. Eng. Manuf. Green Technol..

[4] Garate J., Solovitz S.A., Kim D. (2018). Fabrication and performance of segmented thermoplastic composite wind turbine blades. Int. J. Precis. Eng. Manuf. Green Technol..

[5] (2025). Composites World Report. Composites end Markets: New Space (2025). https://www.compositesworld.com/articles/composites-end-markets-new-space-(2025).

[6] Thomason J.L., Vlug M.A. (1996). Influence of fiber length and concentration on the properties of glass fiber-reinforced polypropylene: Part 1-Tensile and flexural modulus. Composites.

[7] Thomason J.L. (2007). The influence of fibre length and concentration on the properties of glass fibre reinforced polypropylene: Interface strength and fibre strain in injection moulded long fibre PP at high fibre content. Compost. Part A Appl. Sci. Manuf..

[8] Wang C., Yang S. (2013). Thermal, tensile and dynamic mechanical properties of short carbon fibre reinforced polypropylene composites. Polym. Polym. Compos..

[9] Cilleruelo L., Lafranche E., Krawczak P., Pardo P., Lucas P. (2012). Injection moulding of long glass fibre reinforced poly(ethylene terephtalate): Influence of carbon black and nucleating agents on impact properties. Express Polym. Lett..

[10] Rohde M., Ebel A., Wolff-Fabris F., Altstädt V. (2011). Influence of processing parameters on the fiber length and impact properties of injection molded long glass fiber reinforced polypropylene. Int. Polym. Process..

[11] Goris S., Back T., Yanev A., Brands D., Drummer D., Osswald T.A. (2018). A novel fiber length measurement technique for discontinuous fiber-reinforced composites: A comparative study with existing methods. Polym. Compos..

[12] Nguyen B.N., Bapanapalli S.K., Holbery J.D., Smith M.T., Kunc V., Frame B.J., Phelps J.H., Tucker C.L. (2008). Fiber length and orientation in long-fiber injection-molded thermoplastics-Part I: Modeling of microstructure and elastic Properties. J. Compos. Mater..

[13] Bernasconi A., Cosmi F., Hine P.J. (2012). Analysis of fibre orientation distribution in short fibre reinforced polymers: A comparison between optical and tomographic methods. Compos. Sci. Technol..

[14] Gandhi U., Goris S.D.B., Kunc V., Song Y.Y. (2015). Method to measure orientation of discontinuous fiber embedded in the polymer matrix from computerized tomography scan data. J. Thermoplast. Compos. Mater..

[15] Teuwsen J., Goris S., Osswald T.A. (2017). Impact of the process-induced microstructure on the mechanical performance of injection molded long glass fiber reinforced polypropylene. Proceedings of the SPE ANTEC Annual Tech Paper.

[16] Huang C.T., Chu J.H., Fu W.W., Hsu C., Hwang S.J. (2021). Flow-induced orientations of fibers and their influences on warpage and mechanical property in injection fiber reinforced plastic (FRP) Parts. Int. J. Precis. Eng. Manuf. Green Technol..

[17] Quintana M.C., Bernath A., Schajer G., Reiter M., Leitner M., Steinmüller H. (2020). Fiber orientation distribution predictions for an injection-molded Venturi-shaped part validated against micro-CT. Front. Mater..

[18] Folgar F., Tucker C.L. (1984). Orientation behavior of fibers in concentrated suspensions. J. Reinf. Plast. Compos..

[19] Advani S.G., Tucker C.L. (1987). The use of tensors to describe and predict fiber orientation in short fiber composites. J. Rheol..

[20] Vincent M., Giroud T., Clarke A., Eberhardt C. (2005). Description and modeling of fiber orientation in injection molding of fiber reinforced thermoplastics. Polymer.

[21] Ortman K., Baird D., Wapperom P., Aning A. (2012). Prediction of fiber orientation in the Injection molding of long fiber suspensions. Polym. Compos..

[22] Phelps J.H., Tucker C.L. (2009). An anisotropic rotary diffusion model for fiber orientation in short- and long-fiber thermoplastics. J. Non-Newton. Fluid Mech..

[23] Wang J., O’Gara J.F., Tucker C.L. (2008). An objective model for slow orientation kinetics in concentrated fiber suspensions: Theory and rheological evidence. J. Rheol..

[24] Wang J., Jin X. Comparison of recent fiber orientation models in autodesk moldflow insight simulations with measured fiber orientation data. Proceedings of the Polymer Processing Society 26th Annual Meeting (PPS-26).

[25] Tseng H.C., Chang R.Y., Hsu C.H. (2013). Phenomenological improvements to predictive models of fiber orientation in concentrated suspensions. J. Rheol..

[26] Żurawik R., Volke J., Zarges J.-C., Heim H.-P. (2022). Comparison of real and simulated fiber orientations in injection molded short glass fiber reinforced polyamide by X-ray microtomography. Polymers.

[27] Rienesl K., Lengauer C., Jarosch K., Pinter G. (2023). Determination of fiber orientation model parameters for injection-molded short fiber reinforced polymers. Front. Mater..

[28] Libscomb G.G., Denn M.M., Hur D.U., Boger D.V. (1988). The flow of fiber suspensions in complex geometries. J. Non-Newton. Fluid Mech..

[29] VerWeyst B.E., Tucker C.L. (2002). Fiber suspensions in complex geometries: Flow/orientation coupling. Can. J. Chem. Eng..

[30] Tseng H.C., Su T.H. Coupled flow and fiber orientation analysis for 3D injection molding simulations of fiber composites. Proceedings of the Polymer Processing Society 34th Annual Meeting (PPS-34).

[31] Favaloro A.J., Tseng H.C., Pipes R.B. (2018). A new anisotropic viscous constitutive model or composite molding simulation. Compos. Part A.

[32] Tseng H.C., Favaloro A.J. (2019). The use of informed isotropic constitutive equation to simulate anisotropic rheological behaviors in fiber suspensions. J. Rheol..

[33] Huang C.T., Lai C.H. (2020). Investigation on the coupling effects between flow and fibers on fiber-reinforced plastic (FRP) injection parts. Polymers.

[34] Beaumont J.P., Young J.H., Jaworski M.J. (1998). Solving Mold Filling Imbalances in Multi-Cavity Injection Molds. J. Inject. Molding Technol..

[35] Beaumont J.P., Young J.H. (1999). Mold filling imbalances in Geometrically Balanced Runner Systems. J. Reinf. Plast. Compos..

[36] Beaumont J.P. (2004). Runner and Gating Design Handbook.

[37] Chiang G., Chien J., Hsu D., Tsai V., Yang A. True 3D CAE visualization of filling imbalance in geometry-balanced runners. Proceedings of the SPE Annual Tech Meeting (ANTEC2005).

[38] Chien J., Huang C., Yang W., Hsu D. True 3D CAE Visualization of “Intra-Cavity” Filling Imbalance in Injection. Proceedings of the SPE Annual Tech Meeting (ANTEC2006).

[39] Petzold F., Thornagel M., Manek K. Complex Thermal Hot-Runner Balancing: A Method to Optimize Filling Pattern and Product Quality. Proceedings of the SPE Annual Tech Meeting (ANTEC2009).

[40] Wilczyński K., Narowski P. (2020). A Strategy for Problem Solving of Filling Imbalance in Geometrically Balanced Injection Molds. Polymers.

[41] Shotwell D., Román A.J., Yang H., Lin C.-Y., Glas S., Chen E., Osswald T.A., Turng L.-S. (2022). Effect of static mixer on fiber orientation of injection molded fiber-reinforced composites. SPE Polymers.

[42] Land P., Krumpholz T., Heim H.-P. (2022). Comparison of fibre reorientation of short- and long-fibre reinforced polypropylene by injection molding with a rotating mold core. Int. Polym. Process..

[43] Hsieh F.-L., Chen C.-T., Hwang S.-S., Hwang S.-J., Huang P.-W., Peng H.-S., Jien M.-Y., Huang C.-T. (2024). Study on the influence of runner and overflow area design on flow–fiber coupling in a multi-cavity system. Polymers.

[44] Huang C.T., Wang J.Z., Lai C.H., Hwang S.J., Huang P.W., Peng H.S. (2023). Correlation Between Fiber Orientation and Geometrical Shrinkage of Injected Parts Under the Influence of Flow-Fiber Coupling Effect. Int. J. Precis. Eng. Manuf. Green Technol..

